# Supplementary material for: Collaborative intelligence in AI: Evaluating the performance of a council of AIs on the USMLE
Source: PLOS Digit Health. 2025 Oct 9;4(10):e0000787. doi: 10.1371/journal.pdig.0000787 (PMC12510544; doi:10.1371/journal.pdig.0000787)
Supplement: S2 File — (ZIP) [file pdig.0000787.s002.zip › S2 Data Raw data files containing unprocessed question inputs and LLM outputs including transcripts of deliberations/STEP3_Question-61-1_Type-MC-NJ.docx]

**Facilitator** **[Round 1]:** A 35-year-old man comes to the office because of 1-week history of mid low back pain that radiates down his right leg. The pain began after the patient lifted a heavy box onto his truck. He rates his current pain as an 8 on a 10-point scale. He has been unable to find a comfortable position and has been sleeping in a recliner. Medical history is unremarkable and he takes no medications. He has smoked one pack of cigarettes daily for the past 25 years, and he drinks a six-pack of beer on Friday and Saturday nights. BMI is 27 kg/m². He appears uncomfortable and stands during the physical examination. Vital signs are normal. Straight-leg raise test is positive on the right, with loss of right ankle reflex. The remainder of the physical examination discloses no abnormalities. Which of the following is the most likely explanation for this patient’s symptoms? (A) Displacement of the nucleus pulposus (B) Hypertrophy of the facet joints (C) Osteophyte formation (D) Spondylolisthesis (E) Thickening of ligamentum flavum

**AI_C:** RESTATING THE QUESTION: We are talking about a 35-year-old man who is dealing with persistent lower back pain radiating down his right leg, a pain that started after lifting a heavy object. His pain is quite intense, on top of that he is also a long-term smoker and drinks beer on weekends. His BMI indicates that he is overweight. He doesn't find relief in any position and thus has resorted to sleeping in a recliner. His straight-leg raise test was positive on the right side and a loss of right ankle reflex was noted, indicating potential nerve root irritation or compression. The query here is, which abnormal condition best accounts for his symptoms? PLANNING: The potential choices include displacement of the nucleus pulposus, hypertrophy of the facet joints, osteophyte formation, spondylolisthesis, and thickening of the ligamentum flavum. Displacement of nucleus pulposus refers to a scenario where the central portion of an intervertebral disc, the nucleus pulposus, has been displaced or ruptured leading to a herniated disc. Hypertrophy of the facet joints, and thickening of the ligamentum flavum, osteophyte formation and spondylolisthesis mostly occur in older patients with degenerative disc diseases. Therefore, the most clinically relevant concern here seems to be displacement of the nucleus pulposus. CLINICAL REASONING: This patient's symptoms are consistent with lower lumbar disc herniation, specifically, L5/S1 herniation. Lifting heavy weights is a common cause of disc herniation, because it places intense strain on lower back. This is also consistent with positive straight-leg raise test. The lack of a right ankle reflex is another key clue here, as the S1 nerve root, which supplies the gastrocnemius muscle controlling ankle reflex, is commonly affected in L5/S1 disc herniation. CONCLUDING: In patients of this age group, the abrupt onset of radicular back pain (pain that radiates along the path of a spinal nerve root) after an event involving heavy lifting is most commonly due to herniation of a lumbar intervertebral disc. RESPONSE: Therefore, given the patient's symptomatology, including the positive straight-leg raise test and loss of right ankle reflex, displacement of the nucleus pulposus, resulting in a herniated disk, is the most likely explanation for this patient’s symptoms. (Choice A)

**AI_B:** 1) RESTATING THE QUESTION: In simple terms, a 35-year-old male presents with a complaint of severe back and right leg pain that started a week ago when he was lifting a heavy box. His weight, smoking and drinking history indicate a few unhealthy lifestyle habits, and he's been having difficulty finding a position that alleviates his pain. His physical exam suggests a nerve problem on the right side, as indicated by a positive straight-leg raise test and a missing ankle reflex. We need to find out the most probable cause for this man's symptoms among the following possibilities: displacement of the nucleus pulposus, hypertrophy of the facet joints, osteophyte formation, spondylolisthesis, or thickening of ligamentum flavum. 2) PLANNING: The described symptoms and signs might suggest a spinal nerve root compression, considering that the individual exhibits a positive straight-leg raise test and loss of right ankle reflex. The compression could be caused by either a bulged intervertebral disc, a calcified enlarged facet joint, a bone spur of the vertebrae, a moved vertebrae, or a thickened ligamentum flavum. 3) CLINICAL REASONING: An analysis of the patient's history - the pain originated after lifting a heavy box, is severe, radiates down one leg and is accompanied by a positive straight-leg raise test - could point to the disc herniation or displacement of the nucleus pulposus (option A). The hypertrophy of the facet joints or the thickening of ligamentum flavum (options B and E) usually tend to cause neurogenic claudication rather than radiculopathy. Ostheophyte formation (option C) typically causes chronic symptoms rather than the acute onset experienced by this patient. Spondylolisthesis (option D) usually would have other findings such as a palpable step-off in the lower spine and would have been mentioned in the physical examination. 4) CONCLUDING: Given the patient's symptoms, history, and the findings on physical examination, the most probable cause of his condition seems to be displacement of the nucleus pulposus, which can exert pressure on the spinal nerves causing the signs and symptoms presented by the patient. 5) RESPONSE: The most likely explanation for this patient’s symptoms is (A) Displacement of the nucleus pulposus.

**AI_D:** (1) RESTATING THE QUESTION: A 35-year-old male presents with a week-long episode of low back pain, intensified by heavy lifting, and radiates down his right leg. The patient, a heavy smoker and occasional heavy drinker, has been experiencing severe discomfort leading to poor sleep quality. His straight-leg raise test is positive on the right side, and he has experienced a loss of right ankle reflex. We're asked to identify the most likely reason for his symptoms from a list of options that include displacement of the nucleus pulposus, facet joint hypertrophy, osteophyte formation, spondylolisthesis, and thickening of the ligamentum flavum. (2) PLANNING: Given his clinical presentation and test results, we might assume different underlying pathologies, such as a herniated disc, a problem with the intervertebral joints, degenerative changes leading to bone spurs, anterior displacement of a vertebral body, or ligamentous changes. We need to focus on those pathologies that can potentially cause radiating leg pain along with back pain. (3) CLINICAL REASONING: This patient's radicular pain combined with a positive straight-leg raise test and loss of ankle reflex indicates compression or irritation of the L5 or S1 nerve root commonly caused by herniation of the lumbar intervertebral disc; this is evidenced by the "displacement of the nucleus pulposus". This condition, also known as a slipped disc or herniated disc, occurs when the nucleus pulposus, the inner core of the vertebral disc, is forced through the outer fibrous ring (annulus fibrosus) and protrudes into the spinal canal. It can impinge nerve roots, causing symptoms like the ones this patient is presenting. The onset of symptoms following lifting is also typical for disc herniation. (4) CONCLUDING: Other options would likely present with different signs or symptoms. Hypertrophy of the facet joints, osteophyte formation, spondylolisthesis, or thickening of ligamentum flavum, could theoretically lead to back pain, but they are less commonly associated with radiating leg pain, a positive straight-leg raise test, and loss of the right ankle reflex. (5) RESPONSE: Therefore, the most likely explanation for this patient's symptoms is (A) Displacement of the nucleus pulposus, or a herniated lumbar intervertebral disc.

**AI_A:** 1) RESTATING THE QUESTION: The medical inquiry revolves round a 35-year-old male patient who has been suffering from low back pain for a week. This pain, which rates at 8 out of 10 on a scale of pain intensity, radiates down his right leg. The onset of the pain was after lifting a heavy box. He smokes one pack of cigarettes daily and consumes a six-pack of beer during the weekends. His BMI is 27 kg/m², and he has positive right-sided straight-leg raise test with the loss of the right ankle reflex. It is asked what could likely be the cause of his symptoms among the options: displacement of the nucleus pulposus, hypertrophy of the facet joints, osteophyte formation, spondylolisthesis, or the thickening of ligamentum flavum. 2) PLANNING: Key factors to consider are his age, the onset, and nature of the pain (came after lifting a heavy object and radiates down the leg), positive straight-leg raise test and loss of ankle reflex. These symptoms suggest compressive neuropathy, likely due to a herniated intervertebral disc, as this condition commonly causes sciatica-like pain and is typically seen after heavy lifting. 3) CLINICAL REASONING: The straight-leg raise test is a common maneuver used in physical examination to test for lumbar radiculopathy. It is positive when it reproduces the patient's sciatica. Loss of reflexes, such as the ankle reflex, is another feature suggestive of radiculopathy. The displaced nucleus pulposus could compress the nerve root, causing the radiating pain. Although conditions like hypertrophy of facet joints, osteophyte formation, spondylolisthesis, and thickening of ligamentum flavum also cause back pain and radicular symptoms, the typical history of lifting heavy weights, the acute onset of radicular pain, and the straight-leg rise test align more with disc herniation. 4) CONCLUDING: Based on the clinical features of acute onset, radicular pain following lifting of a heavy object, a positive straight-leg raise test along with loss of ankle reflex and no other abnormalities on physical examination, the most likely explanation for this patient’s symptoms would be a displaced nucleus pulposus. 5) RESPONSE: Thus, taking into account all the assumptions and counter-assumptions, the primary cause of this patient's symptoms should be (A) Displacement of the nucleus pulposus.

**AI_E:** RESTATING THE QUESTION: The inquiry involves a 35-year-old man who is experiencing severe pain in his mid-lower back that radiates down his right leg. The pain only became noticeable after he lifted a heavy object onto his truck. His pain level is quite high, reaching an 8 out of 10, on the pain scale. He struggles to find a comfortable position to alleviate his symptoms. His medical history is quite ordinary, and he does not take any medications. He smoked pack of cigarettes daily for twenty-five years and consumes alcohol on the weekends. His Body Mass Index (BMI) is 27 kg/m², showing overweight. During his physical examination, he appears in discomfort and chooses to stand up. His vital signs are within normal parameters. His straight-leg raise test is positive on the right side, accompanied by the loss of the right ankle reflex. All other physical examination findings are normal. We are asked to find the most likely cause of this patient's symptoms among the given options: displacement of the nucleus pulposus, hypertrophy of the facet joints, formation of osteophytes, spondylolisthesis, or thickening of the ligamentum flavum. PLANNING: As an initial assumption, the clinical scenario and the positive result of the straight-leg raise test hint towards nerve compression of some sort due to the radiating pain down the leg and the loss of ankle reflex. Among the options, several could lead to nerve compression but the lifting incident indicates an acute event rather than a chronic condition, which makes the displacement of nucleus pulposus (also known as herniation of an intervertebral disc) more probable. However, we need to keep in mind other factors in this patient's lifestyle that could contribute to chronic issues such as smoking, alcohol consumption, and overweight. CLINICAL REASONING: Lifting heavy objects has long been known as a risk factor for herniation of intervertebral discs due to the sudden surge of pressure. In such a situation, the nucleus pulposus, an inner part of the intervertebral disc, can get displaced and exert pressure on the surrounding nerves causing radiating pain, the positive result of the straight-leg raise test, and loss of reflexes. Other choices might lead to similar symptoms but considering the acute onset of symptoms after a lifting event, disc herniation is more likely as hypertrophy of the facet joints, osteophyte formation, spondylolisthesis, or thickening of the ligamentum flavum, all of which are typically chronic conditions associated with aging, trauma, or arthritis. CONCLUDING: Considering the patient's symptoms, examination findings, the acute onset of pain after lifting the heavy object, the presence of radiculopathy, along with other findings like the positive straight-leg raise test and loss of the right ankle reflex, the most likely explanation for this patient's symptoms is acute herniation of the intervertebral disc due to the displacement of the nucleus pulposus causing pressure on the nerve roots. RESPONSE: Thus, the most likely explanation for the patient's symptoms is (A) displacement of the nucleus pulposus (also known as herniation of an intervertebral disc).

**Facilitator** The consensus among the AI responses is that the most likely explanation for the 35-year-old male patient's symptoms is displacement of the nucleus pulposus, also known as a herniated intervertebral disc. This conclusion is based on the patient's age, the acute onset of his symptoms following a heavy lifting incident, the nature of his pain (severe, radiating down the leg), and the results of his physical examination, which includes a positive straight-leg raise test and loss of right ankle reflex. These symptoms and signs suggest a compressive neuropathy, likely due to a herniated intervertebral disc, which is common after heavy lifting and can cause sciatica-like pain. The other options, such as hypertrophy of the facet joints, osteophyte formation, spondylolisthesis, and thickening of ligamentum flavum, are typically associated with chronic conditions and do not align as closely with the patient's acute symptoms and history.
